# Practitioner perspectives on building capacity for evidence-based public health in state health departments in the United States: a qualitative case study

**DOI:** 10.1186/s43058-020-00003-x

**Published:** 2020-02-25

**Authors:** Stephanie Mazzucca, Cheryl A. Valko, Amy A. Eyler, Marti Macchi, Andrew Lau, Jeanne Alongi, John Robitscher, Ross C. Brownson

**Affiliations:** 1 Prevention Research Center in St. Louis, Brown School, Washington University in St. Louis, One Brookings Drive, Campus Box 1196, St. Louis, MO 63130 USA; 2 National Association of Chronic Disease Directors, Decatur, GA USA; 3 Arcora Foundation, Seattle, WA USA; 4 Department of Surgery (Division of Public Health Sciences) and Alvin J. Siteman Cancer Center, Washington University School of Medicine, Washington University in St. Louis, St. Louis, MO USA

**Keywords:** Evidence-based practice, Public health practice, Workforce development, Chronic disease prevention

## Abstract

**Background:**

Public health agencies are responsible for implementing effective, evidence-based public health programs and policies to reduce the burden of chronic diseases. Evidence-based public health can be facilitated by modifiable administrative evidence-based practices (A-EBPs) (e.g., workforce development, organizational climate), yet little is known about how practitioners view A-EBPs. Thus, the purpose of this qualitative study was to understand state health department practitioners’ perceptions about how A-EBPs are implemented and what facilitators and barriers exist to using A-EBPs.

**Methods:**

Chronic disease prevention and health promotion program staff who were members of the National Association of Chronic Disease Directors were recruited to participate in telephone interviews using a snowball sampling technique. Interviews were transcribed verbatim, and transcripts were analyzed using a common codebook and the a priori method in NVivo.

**Results:**

Twenty seven interviews were conducted with practitioners in four states (5–8 interviews per state). All practitioners felt that their work unit culture is positive and that leadership encouraged and expected staff to use evidence-based processes. Participants discussed the provision of trainings and technical assistance as key to workforce development and how leaders communicate their expectations. Access to evidence, partnerships, and funding restrictions were the most commonly discussed barriers to the use of A-EBPs and EBDM.

**Conclusions:**

Results of this study highlight practitioners’ perspectives on promoting evidence-based public health in their departments. Findings can inform the development and refinement of resources to improve A-EBP use and organizational and leadership capacity of state health departments.

Contributions to the literature

- Implementing evidence-based public health is challenging but can be facilitated at the organizational level using administrative evidence-based practices (A-EBPs).
- In interviews with state health department chronic disease staff, practitioners reported successes (e.g., leaders dedicated to evidence-based public health) and challenges (e.g., forming and maintaining partnerships) to using A-EBPs.
- Knowledge of practitioners’ views of how A-EBPs operate within their SHD can inform development of effective strategies to improve agency capacity that are relevant to practitioners and ultimately improve public health practice.

## Background

The prevalence of and costs associated with chronic disease, including cardiovascular disease, cancer, and diabetes, across the globe are staggering, with an estimated 71% of deaths worldwide due to chronic disease [1]. Recent data suggest that 88% of deaths in 2016 in the United States (US) are attributable to chronic diseases [1], and chronic diseases account for 90% of the $3.5 trillion spent annually on health care [2, 3]. Promotion of health behaviors including healthy eating, physical activity, avoiding tobacco, and participating in cancer screenings can prevent chronic diseases and manage complications of those with chronic diseases [4–8]. Thus, public health systems across the world have been tasked with intervening at the population level to reduce the burden of chronic disease, especially for groups of people experiencing inequities in health due to social determinants of health, such as insurance status, income, education, and the characteristics of environments in which people live (e.g., built environment) [9]. Additionally, many governmental agencies across the world have emphasized the need for public health systems to use evidence-based approaches in their chronic disease prevention efforts [10–13].

Evidence-based public health (EBPH) is an approach to public health practice in which public health practitioners identify, implement, and evaluate evidence-based interventions (EBIs), including those focused on chronic disease prevention. EBPH is characterized by the use of evidence-based decision-making (EBDM), which is the process of integrating the best available research evidence, practitioner expertise, and the characteristics, needs, and preferences of the community [14, 15]. EBDM allows public health practitioners to identify, implement, and evaluate evidence-based programs and policies that are relevant for their communities [14]. There is an ethical justification for using of EBDM [16, 17], and EBDM is associated with the improved use of evidence-based interventions (EBIs) and public health agency performance [18–22]. Additionally, EBDM can facilitate the efficient use of finite resources, as this process helps prioritize implementation of programs and policies that can have the biggest impact on the public’s health given the financial and personnel investments of a public health program (i.e., maximizing cost-effectiveness). Practitioners and researchers have emphasized the use of EBDM to improve public health practice [23–28], and EBDM components are prominent in US Public Health Accreditation Board standards [29]. Therefore, there is a need for efforts to better understand and foster EBDM within public health agencies.

For EBDM to operate efficiently, individual practitioners must have appropriate, relevant skills and the organizations in which they work must have sufficient resources, infrastructure, and leadership [30]. Administrative evidence-based practices (A-EBPs) are structures and activities at the organizational level that can facilitate evidence-based decision-making in health departments, including practices such as provision of skills-based training, leaders setting expectations for EBDM in strategic plans, and allocating financial resources for quality improvement and implementation of programs [23, 31]. A-EBPs can be grouped into five domains: workforce development, organizational climate and culture, leadership, relationships and partnerships, and financial processes. A-EBPs can be positively associated with performance measures, e.g., achieving core public health functions, carrying out evidence-based interventions [32], can potentially be modified over the course of a few years, and are within the control of public health agencies. Thus, understanding how A-EBPs operate within public health agencies and what is needed to support them can inform strategies to build capacity for EBDM within public health agencies.

Three levels of government operate in the United States to conduct public health: national, state, and local. At the national level, the Department of Health and Human Services (DHHS) Agencies with the DHHS, e.g., the Centers for Disease Control and Prevention (CDC), establish funding priorities and distribute public funds to state and local health departments for specific chronic disease prevention and treatment activities (e.g., cancer screening, tobacco control, healthy eating, physical activity promotion) [33]. These agreements often offer some degree of flexibility in how states and local public health agencies use the funds. For example, state agencies can choose from a number of chronic disease prevention strategies provided by CDC, e.g., improving access to healthy food and beverages, and what programs to implement to address a chosen strategy [34]. Often in larger grants, state health departments (SHD) serve as pass-through agencies to distribute funds, largely from DHHS, to contracted partner organizations, e.g., local health departments or community-based organizations. Local health departments, similar to local governments in Australia, provincial public health departments in Canada, and local authorities in the UK [35], are frontline organizations responsible for directly delivering public health programs to their towns or cities.

Within a SHD, chronic disease departments vary widely in terms of the level of hierarchy, specific positions and titles, and how governance is shared with local level agencies [36]. Regardless of the variation, decision-making is complex and shared among several types of practitioners: those in leadership positions (e.g., division directors, program managers) and those in lower-level positions (e.g., health educators). In SHDs, patterns and correlates of A-EBPs have been identified [31], and several studies have focused on awareness of, training for, and differences in A-EBPs in local public health settings [23, 25, 32, 37]. Research about SHD practitioners’ perspectives of the use of A-EBPs is limited, which is essential to support the development of strategies to support the use of A-EBPs that SHD practitioners would find relevant. Thus, the purpose of this study was to understand SHD practitioners’ views about how A-EBPs are implemented and what hinders and supports their use.

## Methods

This paper describes results from a qualitative study designed to understand the perspectives of SHD chronic disease practitioners about A-EBPs and how they relate to EBPH. The larger study from which this data comes was a cross-sectional study using a quantitative survey of SHD practitioners to examine capacity for EBPH in SHDs followed by a qualitative study using a subset of respondents from the quantitative survey.

### State site selection

States were selected as part of a related evaluation of the National Association of Chronic Disease Directors’ (NACDD) efforts to improve evidence-based public health capacity. NACDD is a professional organization dedicated to improving “the health of the public by strengthening state-based leadership and expertise for chronic disease prevention and control in states and at the national level” within the United States [38]. In 2015, a national survey was conducted of state chronic disease directors and state chronic disease prevention and health promotion program staff who were members of the National Association of Chronic Disease Directors [31, 39]. States were ranked by overall capacity in EBPH using participants’ responses to items in the national survey about the use of EBDM in their health department, public health accreditation status (accreditation indicating higher capacity), and the preventable burden of disease in each state. States were not eligible if they had fewer than five respondents to the national survey or were involved with the Prevention Research Center’s STRIDE study [40]. Five respondents to the quantitative survey were needed for a state to be eligible because the study team deemed this enough response to represent the state’s capacity for chronic disease EBPH and not just the capacity of individual practitioners. States involved in the STRIDE study were ineligible because this study focused on capacity building efforts for SHD practitioners, which would likely have influenced practitioners’ reporting of related concepts to the same research team. From the ranking, a total of four state health departments were invited to participate in the study. In order to gain the perspectives of different types of state health departments, two states identified as higher capacity and two identified as lower capacity were included in the sample.

### Interview recruitment

The Principal Investigator of the study emailed the Chronic Disease Director of each of the four states and invited the health department to participate in the study. Between five and eight state chronic disease prevention and health promotion program staff were recruited for the case study per state (four total states) using a snowball sampling technique. All chronic disease prevention or health promotion program staff, i.e., practitioners, in the state health departments of the four sampled states received an invitation to participate in an interview. Staff who were not involved in programmatic decisions or implementing programs were ineligible, e.g., data entry staff. Participants were told that the purpose of the study was to understand their opinions about evidence-based public health in their departments. Interviews occurred between December 2016 and May 2017. Participants who completed the interview were offered a $25 donation to a non-profit organization from a list of options.

### Interview guide development

Interview questions focused on gaining perceptions of evidence-based decision-making (Additional file 1). The research team and NACDD staff developed questions from the initial survey of SHD practitioners and previous research focused on evidence-based public health that was grounded in Diffusion of Innovations and Institutional Theory [40] and Aarons’ conceptual model of evidence-based practice implementation in public service sectors [41, 42]. The interview guide was divided them into four main sections: administrative support for evidence-based programs and policies, organizational support for evidence-based interventions, networks and partnerships to support evidence-based decision-making, and health equity. The focus of this paper is the first three sections, which included questions about access to and relevance of evidence, leaders’ supports and expectations for EBDM, barriers and facilitators to implementing EBIs, types of partnerships, and recommendations for improving organizational supports for EBDM; the health equity data are presented elsewhere [43]. Training for interviewers included the purpose of the study, appropriate interview strategies, and questions on the interview guide. Thematic saturation was considered to be reached once no new categories of information or themes emerged from additional interviews, as decided by the research team [44]. For example, when research team discussions based on field notes indicated that the concepts of formal and informal communication of EBPH by leaders had been developed sufficiently and little new information on this concept was emerging in new interviews, that theme was considered saturated.

### Data analysis

Interviews were analyzed according to qualitative research standards established by Hennink and colleagues [45]. Field notes were made after each call to supplement interpretation of interview coding. Each recorded interview was professionally transcribed. Transcripts were analyzed using NVivo (version 11) using the principles of thematic analysis, which emphasizes using a combination of deductive and inductive approaches to qualitative data analysis [46, 47]. A common codebook (Additional file 2) was developed deductively, i.e., codes that were developed from the interview guide, and inductively, i.e., codes were added as themes emerged from initial coding. Using a constant comparative coding technique [48], transcripts were coded using the initial codebook, and then recoded as additional themes emerged. Research assistants received one-on-one training from a staff member experienced in qualitative coding and were continually coached throughout the coding process. Fifty percent of the transcripts were coded by at least two people to increase consistency (three coders total for the project), and discrepancies were discussed with a third reviewer to come to a consensus on each inconsistency. Coded comments were synthesized into overall themes, and these themes were then further subdivided and categorized in NVivo (version 11). Results are presented by A-EBP domain, i.e., workforce development, leadership, organizational climate and culture, relationships and partnerships, and financial processes. There were no meaningful differences between high and low capacity states (the sampling approach), and the intent was not to examine differences by capacity. Thus, the results are presented overall and not stratified by state capacity. This study was approved by the Washington University in St. Louis Human Research Protection Office, and adherence to qualitative reporting guidelines is provided in Additional file 3.

## Results

A total of 27 telephone interviews were completed to reach thematic saturation, with 5–8 interviews conducted with participants in each state. Interviews ranged from 21 to 77 min in length (mean = 60 min). Chronic disease-related staff at state health departments had been in their current position for an average of 4.4 ± 3.7 years and had been working in public health for an average of 14.2 ± 7.0 years (Table 1).

**Table 1:** Sample characteristics (*n* = 27)

		*n*
Interviews per state		
State 1		8
State 2		5
State 3		6
State 4		8
	Mean	SD
Number of staff supervised	4.4	3.7
Number of people in unit	52.1	118.9
Years in current position	3.6	3.0
Years with organization	9.1	6.2
Years in public health	14.2	7.0

*SD* standard deviation

### Workforce development

Practitioners discussed two components related to workforce development: employees’ training and experience upon hire and ongoing training opportunities.

Formal public health training and/or previous experience with evidence-based health practice were considered critical qualifications for a successful candidate that would be written into a job description formally. Additionally, an openness to learning and valuing EBDM were noted as important characteristics for potential employees that might be more tacit expectations not formally stated within a job description. Practitioners highlighted the importance of these qualities in light of staffing challenges in state and local public health departments, for example high turnover in many agencies.

Once employees are hired, ongoing professional development is delivered most commonly through trainings and webinars. Some participants noted a desire for trainings to help “program managers to think in a way that is … to train them not only in the tools of an evidence-based approach, but also the way to think in terms of not necessarily accepting something because … you’ve always done it, but to look at what the latest is, to promote an environment of active learning.”

Variation in how much training opportunities are used depended on the internal capacity of the SHD and available funds to attend more expensive, external professional development opportunities like in-person conferences. Practitioners reported the availability of external training opportunities from organizations such as CDC and National Association of Chronic Disease Directors, but fewer opportunities internal to their own department. External opportunities for professional development were considered useful by the majority of practitioners and preferable over no opportunities; however, some practitioners expressed a desire to build capacity within their work units to develop internal trainings that can be tailored to the local context of the SHD, the chronic diseases of focus, and the specific professional development needs within the work unit.

### Leadership

Leaders’ commitment to evidence-based work was described as important to the overall functioning and mission of the SHD. One participant noted that “there’s just a pretty high level of commitment to raising the performance of staff [through EBDM] and an understanding that it’s essential to good public health practice.” Participants described formal and informal ways that leadership communicates their expectations for EBPH processes. Formally, leaders institutionalized their expectations for EBPH through strategic plans.

The department has a strategic plan, and evidence-based public health is prominently displayed as an objective in the strategic plan. It’s right up there with employee retention and reducing obesity as one of the main objectives of the department leadership.

Practitioners noted that by choosing to include language about the use of EBPH within these plans, leaders set the tone for the department and make opportunities for employees to use resources and training opportunities to improve their EBPH skills more available. Informal communication about EBPH in meetings, weekly emails, or other in-person interactions reinforced the formal expectations. In this regard, leaders implicitly communicated that there was “just [a] whole mindset change that this is what [the work unit was] going to be doing now. [They’re] not going to do the same old same old, that you will make sure that you have evidence-base for what you’re doing.”

### Organizational climate and culture

Participants noted that their organizational culture supported EBPH. An exemplary quote of this concept was “[the use of EBPH has] sort of reached a level of acceptance [within their work unit] that’s so high that it’s more remarkable for its absence.” The expectation for using EBPH was sometimes discussed in tandem with the expectation of the CDC and/or leadership to use EBPH, indicating that some practitioners viewed EBPH culture created in a top-down fashion. Some practitioners mentioned that the use of EBPH was limited by the lack of dedicated time for strategic planning and lack of evaluation data that is incorporated into strategic planning.

Practitioners expressed that being able to access evidence and use it in planning helps justify programmatic decisions to leaders. This information provides confidence and credibility in presenting information to stakeholders who might need to be convinced of priority issues. Additionally, access to knowledge about EBIs makes it easier for practitioners to “… know what [they’re] going to measure... and to work with [their] local partners to be able to put that in place of, well here are [the] indicators and here’s what [they should be] be measuring and supporting and why.”

Practitioners reported a variety of available resources to access evidence in planning and implementing projects, including the CDC’s Guide to Community Preventive Services (the Community Guide) and resources developed by NACDD and National Association of City and County Health Officials to summarize available EBIs. Primary literature was discussed as an important source of cutting-edge evidence; however, participants noted that digital access to literature is limited due to subscription fees and lack of knowledge on how to navigate digital libraries. Several mentioned digital library projects and resource banks and having student interns with access to journals through their educational institutes as ways of facilitating access to digital resources.

Many practitioners highlighted the importance of access to evidence that is relevant to the populations they serve. Practitioners considered the available evidence mostly relevant to their work and the populations they serve. When there is not a direct fit, they focus on balancing fidelity with fit when selecting and implementing EBIs. There was a suggestion that practitioners “can look at [an EBI] in terms of like this is the other state, so to apply that here in [our state], you always have to kind of tailor it for that context.” Once an EBI has been tailored, practitioners noted that they can ensure fit within their community by working with their community members to pilot test materials and programs before implementation and with considering evidence in conjunction with local data. Participants often noted that evidence was not relevant for certain populations, such as different racial or ethnic groups or rural communities. Additionally, when practitioners are working within a pre-defined subset of the population, such as people with disabilities, there is sometimes too much variation within the subset of the population to know whether an EBI is applicable to the whole subset:

When we’re talking about sometime like … chronic disease self-management programs for people with disabilities, and the disabilities definition is such a huge spectrum, that this evidence-based program might not be effective for some disability populations.

### Relationships and partnerships

Partnerships were described as key to a SHD’s ability to deliver EBIs to communities within their states, and the majority of partnerships described were with public sector organizations. Many of the partners were organizations in health-related sectors, such as medical providers, community-based organizations, and local public and tribal health agencies. However, several practitioners discussed the importance of partnerships in sectors not related to health, including religious organizations, higher education settings, and the state transportation department.

We kind of acknowledge that if we want to drive towards improved health outcomes we’re not going to be able to do that ourselves. We’d have to look beyond our direct spheres of influence and try to partner with other sectors. Other sectors are actually going to have to potentially more of an impact on health outcomes than we can working alone in just our agency.

The connections were either developed organically, e.g., a partner organization and SHD are introduced based on mutual interest and create a project together, or by deliberately seeking out specific partners, e.g., a SHD reaches out to a community organization based on the particular needs of an existing project. Forming partnerships intentionally is facilitated by having a dedicated person who establishes and maintains collaborations. As one participant described,

… I just started to read a lot about the links between air quality and particulate air matter with cardiovascular disease and diabetes. So … I just called over … we have an air quality division that’s in another building, like, can we meet? Can you tell me a little bit about where your hot spots are? And can we map those against where our hot spots are as far as heart failure? And there’s got to be some … a fair amount of overlap. And so I opened up a couple of opportunities for us to look at working particularly in those geographic areas.

Practitioners offered many recommendations for effectively establishing and maintaining partnerships. They should be thoughtfully developed with careful understanding of the audience and context of the partnering organization and community. Listening to and acknowledging the history of potential partners can help develop trusting and respectful relationships and frame discussion of the importance of EBPH.

… When you understand the history that helps us better frame the importance of these evidence-based processes, whereas if you’re not hearing deeply and being thoughtful and understanding that community’s history, it sounds like another government agency coming in and telling you how to live your life.

Additionally, buy-in and mutual agreement between partners and leadership was viewed as a supportive factor in implementation. Strategic planning should guide which partnerships are prioritized, as well as guide which partners have a shared goal or interest and can also benefit from the partnership. Negotiating what the common ground is may take time, humility, and patience.

… There may be health voluntary agencies that are very interested in addressing obesity. Their chosen approach has been … [for schools to] … conduct physical education. And that’s been their issue. That may not necessarily be in concert with the education world around and they’re trying to balance a number of things. As public health, we may be pushing like, yes, we like the idea of physical education. We can assess, based on political feasibility and readiness, but that’s not where education wants to go. But what we can support is improving walkability to and from school to get those physical activity needs met.

Participants discussed that once a partnership is formed, it is important to be set expectations from the beginning, be reliable, and stay in regular communication with partners. However, communication should be intentional; respectful and efficient use of time is needed and having meetings just for the sake of having meetings may be detrimental.

Time, funding, and politics were identified as major barriers to forming and maintaining partnerships. The time it takes to build and maintain relationships can be too much for some departments to take on given competing interests within the department; in this case, quality over quantity may work better for some organizations. Some practitioners voiced that “… It is okay [to] have a small number of partnerships. What we need to achieve is the outcome, the policy, system and environmental change strategies. And if we can do that with five, then who cares that we didn’t have 50?”

Also, forming partnerships may be challenging due to differing politics of potential partner organizations.

[In our state] there’s a strong element of libertarianism, and even if something is evidence-based, … it’s shown to save lives, it’s shown to be economical and saves the state money, you can always weaken support for it [with partners] saying, well that’s just a nanny state.

Last, funding needs to be available to support partners’ projects. Participants explained that this is often not the case, due to lack of flexible funding streams to support partner organizations. One participant noted that an existing partnership had to end because of a change in funds from those that would support personnel costs and direct program costs to funds that would only support direct program costs. As a result of this partnership ending, the program ended because there was no partner organization who could take on program implementation without financial support for personnel.

### Financial characteristics of the agency

Most participants discussed funding, which the authors consider an agency-level characteristic, as it relates to EBI implementation. There was very little discussion of other aspects of finances, e.g., allocating resources for quality improvement. Funding was the top barrier to implementing EBIs that was mentioned by participants, and funding constraints were mentioned in relation to many of the other A-EBP domains. Funding was discussed in terms of the lack of total funding, restrictions on the way funding could be used, and bureaucracy related to funding. Practitioners described funding as a strong influence on the choice of EBI, noting “… funding pretty much drives everything. So if there’s funding that’s available and it’s … tied to a certain evidence-based strategy or program or intervention, … you will follow that.”

Practitioners expressed a desire for flexibility and creativity in gaining and using funding to address the specific needs of communities in their state. Additionally, participants noted they were sometimes frustrated with the way funding decisions are made in state legislatures. Instead of prioritizing funding based on a population health perspective, long-term funding sources are sometimes appropriated to address rare diseases based on vocal advocates such as when “… someone’s appropriated a certain amount of funds forever on topic X because a mom whose kid had [a] condition got a bill passed that sets aside dollars forever related to that. And even if the condition, you know that you have one case a year in your whole state, and you really could better spend that money on something else, there’s nothing you can do about it.”

## Discussion

This study gained important insight into state health department practitioners’ perceptions of A-EBPs, which can influence the use of EBDM. Practitioners in all states reported that their work unit culture is supportive of EBPH processes and that leadership encouraged and expected staff to use evidence-based processes. Participants discussed the provision of trainings and technical assistance as key to workforce development and how leaders communicate their expectations. Access to evidence, partnerships, and funding restrictions were consistently discussed as barriers to the use of A-EBPs and EBDM. Findings from this study can be used to inform the development and refinement of efforts to improve the use of A-EBPs and organizational capacity of state health departments. Making changes at the organization level can be difficult, slow, and costly and involves buy-in from many stakeholders at multiple levels within organizations in complex public health systems [20, 32, 49, 50]. To this end, these interviews identified aspects of the organization that practitioners believe can make a difference to their work unit.

Many opportunities exist to increase access to evidence relevant for public health decision-making. Increasing access to evidence has been a cornerstone of efforts across the globe to improve EBDM, known as evidence-informed decision-making in settings outside of the United States. Organizations have been established to synthesize and translate evidence to public health decision makers in Canada [51], Australia [52], and the UK [13], and these organizations have developed and implemented training for accessing evidence (discussed in the next paragraph). Some practitioners use “grey” literature because peer-reviewed documents are behind journal paywalls [53]. To address this, the movement towards open access journals can facilitate the reach of peer-reviewed literature to practitioners, including open access fees that authors pay to freely distribute articles [54]. One promising strategy to increase access to evidence that is emerging in the Unites States is the academic health department (AHD) partnership, which is a formal or informal arrangement between an academic institution and a governmental public health agency providing mutual benefits in teaching, research, and practice [55–57]. Practitioners within AHD partnerships often have appointments within the academic setting and can therefore enhance their access to resources such as peer-reviewed articles using university credentials. Linkages with academics might allow practitioners to access information and library services provided by university librarians, which Peirson and colleagues noted as important along with accessing peer-reviewed literature [58]. Promoting and institutionalizing these partnerships as part of several Council for Education on Public Health accreditation standards for American schools and programs of public health would ensure broad and sustainable impact on the ability of practitioners to access evidence [59].

Training and leadership development are needed to build skills of the public health workforce [60, 61]. Based on the results of this study, training topics could include information on accessing the literature and adapting EBIs so that they are relevant to the community. Previous and ongoing studies have demonstrated the benefit of different EBDM training approaches for individuals’ confidence and skills related to EBDM [35, 62–67]. Within the United States, there are several training programs and materials available to public health practitioners, including those developed by the Regional Public Health Training Centers, the National Network of Public Health Institutes, the Public Health Foundation, CDC, universities, and professional groups such as the National Association of Chronic Disease Directors (e.g., STAR program). There is a reciprocal relationship between individuals, including leaders, and implementation climate [30, 68–72]; thus, capacity for EBPH at the organizational level might be improved by increasing the capacity of those in leadership positions level in addition to the organizational level. Based on the findings in this study, key topics for leadership training include strategic planning, effective communication, and working with other leaders and lower level employees. Few trainings and interventions exist to directly change the organizational level factors (i.e., A-EBPs) [58, 62], which is likely important to implement in conjunction with individual-level trainings.

Strong leadership is crucial for successful EBPH, as leaders set the tone for organizational climate and culture [19, 20, 73–78]. In turn, employees interpret the culture as what management values and supports [79]. Leaders at multiple levels (e.g., deputy directors, division directors, program managers) play a specific role in promoting a culture of EBPH but need to work together to communicate a consistent message to employees and facilitate EBPH use [79, 80]. As discussed in these interviews and in previous literature, there are many opportunities for leaders to facilitate the use of EBPH. Leaders can influence the implementation climate by role modeling and coaching effective use of EBPH [79]. Additionally, leaders can improve EBDM use communication of clear expectations for using EBPH, securing time and funds for employees to use EBDM, and including EBDM in strategic plans [58, 81]. The influence of a leader may differ by level; for example, Birken and colleagues identified specific roles of middle managers (e.g., program managers), including mediating between strategy and day-to-day operations [68].

Creating and maintaining partnerships was also described as key to successful EBDM and EBI implementation but an area that could be improved. This is especially important for state health departments or other agencies in a similar role, who rely on contracts with partner organizations to directly implement programs within a local community. In particular, forming non-traditional partnerships, including those with private sector organizations, holds promise to improve public health [82]. Regardless of the sector, static resources, e.g., The Community Tool Box’s Creating and Maintaining Partnerships toolkit, exist to assist practitioners in strengthening and maintaining partnerships [83, 84]. Wider dissemination of these existing materials is needed to improve their reach and use. Resources such as interactive materials or trainings may help identify non-traditional partners, develop a common goal or mission, and create effective communication strategies with partners. Developing skills to interact with other stakeholders and organizations is nuanced and may require a more active approach than passive materials such as toolkits, for example through hands-on experience with practice-based research networks [85]. This should be integrated in practitioner training priorities.

The findings of this study are not without limitations. The relatively small sample in this study may limit its generalizability to other public health agencies in the US or globally. Also, the data gathered were the opinions of the staff members and may not represent the entire state health department. The responses from the staff members may have been subject to social desirability bias. Despite these limitations, these results are important for understanding what practitioners themselves think of the role of A-EBPs in promoting EBPH and their needs to improve capacity.

## Conclusions

Future studies and practice-based work is needed to understand the best ways to translate this information into programs, practices, and policies that can improve public health departments’ use of EBPH. As discussed in these interviews and found in previous literature, an approach for improving public health capacity that is tailored to the specific organizational structure and context is likely to be most effective [30]. Additionally, modifications to funding, e.g., reducing restrictions on how funding can be used to implement evidence-based interventions, are needed to allow practitioners to effectively implement programs that can improve the public’s health and wellbeing.

## Supplementary information

**Additional file 1.** Interview guide

**Additional file 2.** Coding Tree

**Additional file 3.** Reporting Checklist

## Data Availability

Please contact the lead author for more information.

## Abbreviations

A-EBP: Administrative evidence-based practices
CDC: Centers for Disease Control and Prevention
EBDM: Evidence-based decision-making
EBI: Evidence-based intervention
EBPH: Evidence-based public health
NACDD: National Association of Chronic Disease Directors
SHD: State health department
