# Supplementary material for: Practitioner perspectives on building capacity for evidence-based public health in state health departments in the United States: a qualitative case study
Source: Implement Sci Commun. 2020 Feb 25;1:34. doi: 10.1186/s43058-020-00003-x (PMC7427867; doi:10.1186/s43058-020-00003-x)
Supplement: Supplementary file 1 — Additional file 1. Interview guide [file 43058_2020_3_MOESM1_ESM.docx]

**Supplemental File 1.** Interview guide

Part 1: Administrative support for evidence-based programs/policies

The first set of questions ask about your work unit’s **support** of the use of an evidence-based process to plan and carry out public health services, programs, and policies.

1.1 How would you describe your work unit culture as it relates to supporting the implementation of evidence-based processes?

Probe:

*How much support do you feel your work unit provides for the processes necessary for utilizing evidence-based programs/policies*?

1.2 What kind of access do you have to existing research evidence for evidence-based processes?

Probes:

*Print (e.g. Academic journals, Reports to funders, Press releases, Newsletters, Policy briefs, Email alerts, Targeted mailings)*

*Trainings or meetings/conferences (e.g. Academic conferences, Seminars or workshops (phone, webinars, or in-person), Face-to-face meetings with stakeholders), Professional associations)*

*Media (e.g. Media interviews, CD-ROMs, Social Media (Facebook, Twitter))*

1.2.A How relevant is this research to the community you serve?

1.3 What types of supports do leaders in your agency provide for evidence-based processes?

Probe:

*For whom the support is provided?*

*How often?*

1.3.A In what ways do leaders in your agency communicate expectations for use of evidence-based processes?

1.4 When thinking about an evidence-based process, what type of supports for evidence-based process are evident in your agency? What types of support are most useful? Which are less useful?

1.5 If you were able to change one thing related to the culture of your work unit in supporting evidence-based processes, what would it be? Why?

Probes:

*Training*

*Better access to relevant materials/information*

*Making it a higher priority*

1.6 When your unit in the agency is hiring employees, what qualifications does your section seek in employees to be sure they can carry out evidence-based processes? Why do these qualifications matter to you?

Probes:

*Public health training*

*A commitment to our mission*

*Specific skill sets*

Part 2: Organizational support for evidence-based interventions

Next, I will be asking you questions the support for evidence-based interventions. Please note that we will be moving away from the evidence-based processes that we have been discussing. We are now discussing **Evidence-based interventions,** which are programs and policies with evidence (based on published research) of improving health. In this instance, we’re referring to the opinions and actions of your work unit/agency and whether these are currently impacting your use of evidence-based interventions. These definition can also be found on your information sheet.

2.1 Think about the set of evidence-based interventions your work unit promotes. What has helped support implementation of these policies and programs? Please describe the process.

2.1.A What helped you to support the implementation of this intervention?

Probes:

*Funds*

*Adequate staff*

*Support from administrators/mangers within your health department*

*Support from elected officials*

*Existence of/partnerships with coalitions*

*Mandates*

*Training*

*Examples from other states*

*Toolkits*

2.2 What are some roadblocks to implementing evidence-based interventions?

2.2.A What are the barriers?

Probes:

*Such as familiarity/knowledge about EBIs*

*Support within your department*

*Financial/staff resources*

*Cost effectiveness information*

*Training*

*Experience*

*Knowledge*

*Limited evidence in some program areas*

*Lack of evidence in some population groups*

*Need to adapt EBIs for cultural appropriateness*

2.2.B How does your agency address the barriers you’ve described?

2.3 What factors contribute to your work unit’s ability to sustain an evidence-based intervention?

Probes:

*Funds*

*Engaged partners*

*Adequate staff*

*Support from policy makers*

*Support from leadership*

*Evaluation capacity*

2.4 What makes it difficult to end an intervention that is not effective?

Probes:

*Funds*

*Engaged partners*

*Adequate staff*

*Support from policy makers*

*Support from leadership*

*Evaluation capacity*

Part 3: Networks and partnerships to support evidence-based decision making

The next questions ask about networks and partnerships to support evidence-based processes. Collaborative **partnerships** (people and organizations from multiple sectors working together in common purpose) are a prominent strategy for community health improvement. This definition can also be found on your information sheet.

3.1 Who does your work unit collaborate with in other health-related sectors (e.g., medical providers, hospitals)?

Probe:

*How were these relationships developed and maintained?*

*What has led to your most successful partnerships?*

3.2 Who does your work unit collaborate with outside of health sectors (e.g., parks & recreation departments, schools, chamber of commerce, faith-based organizations)?

Probe:

*What has led to your most successful partnerships?*

*How were these relationships forged?*

*What has been particularly important for partnerships outside the health sector?*

3.3 What are the barriers for increasing partnerships and collaboration?

Part 4: Health Equity

Many health departments are paying attention to a variety of issues related to **health equity** (when every person has the opportunity to attain his or her full health potential and no one is disadvantaged from achieving this potential because of social position or other socially determined circumstances). We have a few questions about health equity initiatives in your health department.

4.1 What are some examples of programs in your department that have been effective in addressing health equity?

4.1.A which programs are these? Why have they been successful?

Probe:

*Describe how your department ensures that programs address health equity.*

4.2 What steps could be taken within your work unit to better address health equity?

Probes:

*Training*

*Better access to relevant materials/information*

*Making it a higher priority*

4.3 Do you know some ways in which your previously identified partners are addressing health equity? What are they?

4.3.A *What steps could be taken with your partners to better address health equity?*

Probes:

*Training*

*Better access to relevant materials/information*

*Making it a higher priority*

*Putting it in contracts*

*Building relationships with tribes and organizations serving priority populations*

4.4 Often programs in public health occur in silos (disease or risk factor programs with categorical funding). What are some ways to address health equity that would allow us to better cross these silos?

Part 5: Demographics

Thank you. Before we close, we have a few quick questions about your background that we’ll only use in summary form across people interviewed, to summarize in our reports.

5.1 Do you supervise staff? If yes, roughly how many?

5.2 How many people are in your unit?

5.3 How long have you been in your current position?

5.4 How long have you been with this agency or organization?

5.5 How long have you worked in public health overall?
