# Supplementary material for: Practitioner perspectives on building capacity for evidence-based public health in state health departments in the United States: a qualitative case study
Source: Implement Sci Commun. 2020 Feb 25;1:34. doi: 10.1186/s43058-020-00003-x (PMC7427867; doi:10.1186/s43058-020-00003-x)
Supplement: Supplementary file 2 — Additional file 2. Coding Tree [file 43058_2020_3_MOESM2_ESM.docx]

**Supplemental File 2.** Coding Tree

| Node | Code Name | Description | Relating Question(s) |
| --- | --- | --- | --- |
| 1.0 Administrative support for evidence-based programs/policies | | | |
| 1.1 | Culture |  | 1.1 How would you describe your work unit culture as it relates to supporting the implementation of evidence-based processes? |
| 1.1.1 | Funding | Funding requirements impact use and interest in using EBP |  |
| 1.1.2 | Support | Tangible supports for EBP, including training on EBPH, Quality improvement, frameworks, strategic plans, internal support, external support or resources (Community Guide) |  |
| 1.1.3 | Barriers | Barriers or lack of support for EB processes |  |
| 1.1.4 | Great quote | Great quote related to culture |  |
| 1.1.5 | General Culture | Describes the general work unit culture in relation to evidence-based processes, including institutionalization |  |
| 1.2 | Access to Evidence |  | 1.2 What kind of access do you have to existing research evidence for evidence-based processes? |
| 1.2.1 | Frequency of Access | How frequently evidence is accessed |  |
| 1.2.2 | Barriers to Accessing | Barriers to accessing evidence, including lack of staff capacity, lack of access, lack of evidence |  |
| 1.2.3 | Great quote | Great quote related to access to evidence. |  |
| 1.2.4 | General Access to Evidence | Discussion of types of research accessed, through what means (databases, etc.), |  |
| 1.3 | Relevance of Evidence |  | 1.2.A How relevant is this research to the community you serve? |
| 1.3.1 | General Relevance of Evidence | Discussion of relevance of evidence accessed to community being served |  |
| 1.3.2 | Great quote | Great quote related to access to relevance of evidence. |  |
| 1.4 | Leadership Emphasis |  | 1.3 What types of supports do leaders in your agency provide for evidence-based processes? |
| 1.4.1 | Changes over time |  |  |
| 1.4.2 | Great quote | Great quote related to leadership emphasis (or lack thereof) on evidence-based processes. |  |
| 1.4.3 | General Leadership Emphasis | The emphasis and supports (or lack thereof) that leadership has regarding evidence-based processes. |  |
| 1.5 | Communication of Expectations |  | 1.3.A In what ways do leaders in your agency communicate expectations for use of evidence-based processes? |
| 1.5.1 | Externally-based | Communications are a result of externally-based requirements E.g., funding, accreditation |  |
| 1.5.2 | Varies by Organization Structure | The communication of expectations varies by organization structure, such as department. |  |
| 1.5.3 | Great quote | Great quote related to the existence and extent of leadership communication regarding evidence based processes |  |
| 1.5.4 | General Communication of Expectations | The existence and extent of leadership communication regarding evidence based processes |  |
| 1.6 | Type and Quality of Supports |  | 1.4 When thinking about an evidence-based process, what type of supports for evidence-based process are evident in your agency? |
| 1.6.1 | Most Useful |  | What types of support are most useful? |
| 1.6.2 | Less Useful |  | Which are less useful? |
| 1.6.3 | Great quote | Great quote about the types and quality of supports that are most evident in the agency |  |
| 1.6.4 | General type and quality of supports | The types and quality of supports that are most evident in the agency |  |
| 1.7 | Desired Changes to Culture |  | 1.5 If you were able to change one thing related to the culture of your work unit in supporting evidence-based processes, what would it be? Why? |
| 1.7.1 | Great quote | Great quote about desired changes to the culture regarding support when using evidence-based processes. |  |
| 1.7.2 | General Desired Changes to Culture | Desired changes to the culture regarding support when using evidence-based processes. |  |
| 1.8 | Qualifications for Employees |  | 1.6 When your unit in the agency is hiring employees, what qualifications does your section emphasize in employees to be sure they can carry out evidence-based processes? Why do these qualifications matter to you? |
| 1.8.1 | Rationale for Emphasized Qualifications |  |  |
| 1.8.2 | Great quote | Great quote about description of emphasized qualifications when hiring employees. |  |
| 1.8.3 | General Qualifications for Employees | Description of emphasized qualifications when hiring employees. |  |
| 1.9 | Other | For CO, staff capacities | No question (not for NVivo)_ |
| **2.0 Organizational support for evidence-based interventions** | | | |
| 2.1 | Implementation supports | Work-unit, department, and agency support for implementing evidence-based interventions | 2.1 Think about the set of evidence-based interventions your work unit promotes. What has helped support implementation of these policies and programs? |
| 2.1.1 | Great quote |  |  |
| 2.1.2 | General support | Support for EB Interventions | Funding, community champion, policy, etc. |
| 2.2 | Barriers to Implementation | Barriers that are faced when implementing evidence-based interventions | 2.2 What are some roadblocks to implementing evidence-based interventions? |
| 2.2.1 | Biggest Barriers | Code as 2.2.4 – General Barrier |  |
| 2.2.2 | Ideas to Overcome | Code as 2.2.4 – General Barrier |  |
| 2.2.3 | Great quote |  |  |
| 2.2.4 | General Barrier | Any barrier relating to implementation of EB Intervention | Time, staff, funding, data, etc. |
| 2.3 | Agency response to barriers | Work-unit, department, and agency response or lack of response to barriers | 2.2.A How does your agency address the barriers you’ve described? |
| 2.3.1 | Resistance to Address | Code as 2.3.5 – General Response |  |
| 2.3.2 | Ideas for how to Address | Code as 2.3.5 – General Response |  |
| 2.3.3 | Great quote |  |  |
| 2.3.4 | Staff Qualifications | Code as 2.3.5 – General Response |  |
| 2.3.5 | General response | Agency response to barriers when considering EB interventions | Good communication, partnerships, leverage resources, etc. |
| 2.3.6 | Potential Suggestions | Potential suggestions or future directions for agency to consider, to respond to the barriers | (new code , inserted on July 13^th^) |
| 2.4 | Sustainability | Discussion of factors related to sustaining or maintaining evidence-based interventions. These can be factors that act as facilitators or barriers to the sustainability of EBIs and includes reasons for ending effective interventions. | 2.3 What factors contribute to your work unit’s ability to sustain an evidence-based intervention? |
| 2.4.1 | Great quote |  |  |
| 2.4.2 | General Sustainability | General factors contributing to sustainability | Funding, staff, partners, etc. |
| 2.5 | Ending ineffective interventions | Discussion of factors that impact the ability to end an intervention. These can be facilitators or barriers to ending an ineffective intervention. | 2.4 What makes it difficult to end an intervention that is not effective? |
| 2.5.1 | Great quote |  |  |
| 2.5.2 | General ending ineffective intervention | General factors that impact the ability to end an intervention | Losing partners, contractual obligations, etc |
| **3.0 Networks and partnerships to support evidence-based decision making** | | | |
| 3.1 | Health-related partners | List of health-related partners and types of partners, discussion of successful partnerships and how forged | 3.1 Who does your work unit collaborate with in other health-related sectors? What has led to your most successful partnerships?  What has been particularly important for partnerships outside the health sector? How were these relationships forged? |
| 3.1.1 | Great quote |  |  |
| 3.1.2 | General Health Partners | Types of partners | Local health department, associations, medical providers, Medicaid, etc. |
| 3.1.3 | Developed/Maintained | How these relationships developed or maintained | Time, trust, networking, incentives |
| 3.1.4 | Success | What makes the most successful health related partnerships | Constant communication, funding, formal agreement, etc. |
| 3.2 | Outside of Health Sectors | List of partners outside of the health sector and types of partners, discussion of successful partnerships and how forged | 3.2 Who does your work unit collaborate with outside of health sectors? What has led to your most successful partnerships?  What has been particularly important for partnerships outside the health sector? How were these relationships forged? |
| 3.2.1 | Great quote |  |  |
| 3.2.2 | General outside health | Types of partnerships outside health sector | Education system, state department, YMCA, parks and rec, etc. |
| 3.2.3 | Developed/Maintained | How these relationships developed or maintained | Networking, common interest, etc. |
| 3.2.4 | Success | What makes the most successful non-health related partnership | Communication, resources, common goal, etc. |
| 3.2.5 | Importance outside health sector | Why engage in these partnerships | Increase awareness, funding expectations, pool resources, etc. |
| 3.3 | Barriers to Collaboration | Barriers to collaboration and partnerships with those in and out of the health sector | 3.3 What are the barriers for increasing partnerships and collaboration? |
| 3.3.1 | Great quote |  |  |
| 3.3.2 | General Barrier to Collaboration | General barrier to collaboration | Lack of resources, competing agendas, etc. |
| **4.0 Health Equity** | | | |
| 4.1 | Health equity initiatives | Description of efforts—or lack of efforts--to impact health equity. Includes facilitators and barriers | 4.1 What are some examples of programs in your department that have been effective in addressing health equity? 4.1.A Why have these programs been successful? |
| 4.1.1 | Great quote |  |  |
| 4.1.2 | General | Tangible supports to ensure health equity | Mission statement, trainings, strategic plan, etc. |
| 4.1.3 | Barriers to health equity | Barriers to health equity | funding |
| 4.2 | Future steps to address health equity | Thoughts around how the work unit could address health equity differently in the future | 4.2 What steps could be taken within your work unit to better address health equity? |
| 4.2.1 | Great quote |  |  |
| 4.2.2 | General Future | Ways to improve H.E. within department | Access to info, training, increase capacity |
| 4.3 | Partners: Addressing health equity | How partners currently address health equity | 4.3 Do you know some ways in which your previously identified partners are addressing health equity? What are they? |
| 4.3.1 | Great quote |  |  |
| 4.3.2 | General Partners | Partner Tangible support to ensure H.E. | Mission statement, training, etc. |
| 4.4 | Partners: Future steps to address health equity | Thoughts around how partners could address health equity differently in the future | 4.3.A What steps could be taken with your partners to better address health equity? |
| 4.4.1 | Great quote |  |  |
| 4.4.2 | General Future | Ways for partners to improve H.E. | Sharing info, training, CLAS, etc. |
| 4.5 | Breaking silos | How silos impact health equity efforts and how health equity efforts impact silos | 4.4 What are some ways to address health equity that would allow us to better cross these silos? |
| 4.5.1 | Great quote |  |  |
| 4.5.2 | General silos | Ways to address health equity that would allow to better cross silos | Communication, pooled resources, etc. |
| **5.0 Demographics** | | | |
| 5.1 | Staff Supervised |  | 5.1 Do you supervise staff? If yes, roughly how many? |
| 5.2 | Number in unit |  | 5.2 How many people are in your unit? |
| 5.3 | Length of time in position |  | 5.3 How long have you been in your current position? |
| 5.4 | Length of time with organization |  | 5.4 How long have you been with this agency or organization? |
| 5.5 | Length of time in public health |  | 5.5 How long have you worked in public health overall? |
| **6.0 Additional Information** | | | |
| 6.1 | Additional information | Description of any additional information relevant to case study. |  |
| 6.1 | Context |  |  |

| **7.0** | **Other** | Responses that do not fit with another questions. | If new code is occurring within this category, name it as suck |
| --- | --- | --- | --- |
| **NC** | **No Code** | Any text that is not important to keep and therefore, does not need to be coded |  |
