# Supplementary material for: Practitioner perspectives on building capacity for evidence-based public health in state health departments in the United States: a qualitative case study
Source: Implement Sci Commun. 2020 Feb 25;1:34. doi: 10.1186/s43058-020-00003-x (PMC7427867; doi:10.1186/s43058-020-00003-x)
Supplement: Supplementary file 3 — Additional file 3. Reporting Checklist [file 43058_2020_3_MOESM3_ESM.docx]

**Supplemental File 3.** Reporting Checklist

Reference: Tong A, Sainsbury P, Craig J. Consolidated criteria for reporting qualitative research (COREQ): a 32-item checklist for interviews and focus groups. International journal for quality in health care. 2007 Dec 1;19(6):349-57.

| **Item** | **Guide questions/description** | **Answer for our study** |
| --- | --- | --- |
| **Domain 1: Research team and reflexivity** | | |
| *Personal Characteristics* | | |
| Interviewer/facilitator | Which author/s conducted the interview or focus group? | Andrew Lau, MPH |
| Credentials | What were the researcher’s credentials? E.g. PhD, MD | Stephanie Mazzucca, PhD  Cheryl A. Valko, MPH, RD  Amy A. Eyler, PhD, CHES  Marti Macchi, MEd., MPH  Andrew Lau, MPH  Ross C. Brownson, PhD |
| Occupation | What was their occupation at the time of the study? | At the time of the study, occupations were:  SM: Doctoral Student  CV: Project Manager  AE, RB: Professor  MM: Director of Programs  AL: Research Assistant |
| Gender/ | Was the researcher male or female? | The interviewers were male and female (1 each). |
| Experience and training | What experience or training did the researcher have? | Training for interviewers included the purpose of the study, appropriate interview strategies, and questions on the interview guide |
| *Relationship with participants* | | |
| Relationship established | Was a relationship established prior to study commencement? | No relationship was established with participants prior to their interview. |
| Participant knowledge of the interviewer | What did the participants know about the researcher? e.g. personal goals, reasons for doing the research | Participants were told that the purpose of the study was to understand their opinions about evidence-based public health in their departments. |
| Interviewer characteristics | What characteristics were reported about the interviewer/facilitator? e.g. Bias, assumptions, reasons and interests in the research topic | No characteristics are reported about the interviewer or research team. |
| **Domain 2: Study design** | | |
| *Theoretical framework* | | |
| Methodological orientation and Theory | What methodological orientation was stated to underpin the study? e.g. grounded theory, discourse analysis, ethnography, phenomenology, content analysis | Content analysis was used for this study. |
| *Participant selection* | | |
| Sampling | How were participants selected? e.g. purposive, convenience, consecutive, snowball | States were chosen based on state health department practitioners’ reporting of the overall capacity to conduct evidence-based public health on a national survey (purposive sampling). Between five and eight state chronic disease prevention and health promotion program staff were recruited for the case study per state (4 total states) using a snowball sampling technique. |
| Method of approach | How were participants approached? e.g. face-to-face, telephone, mail, email | Participants were approached using a combination of email and telephone contact. |
| Sample size | How many participants were in the study? | N=27 |
| Non-participation | How many people refused to participate or dropped out? Reasons? | We did not track this information. |
| *Setting* | | |
| Setting of data collection | Where was the data collected? e.g. home, clinic, workplace | Interviewers collected data at the workplace; no information was collected about where interviewees took the interview calls. |
| Presence of non-participants | Was anyone else present besides the participants and researchers? | No one else was present in the room with the researchers; no information was collected on who else was in the room with participants. |
| Description of sample | What are the important characteristics of the sample? e.g. demographic data, date | Characteristics of the sample are provided in Table 1. Interviews took place from December 2016 to May 2017. |
| *Data collectio*n | | |
| Interview guide | Were questions, prompts, guides provided by the authors? Was it pilot tested? | Questions and prompts are provided in Supplemental File 1. |
| Repeat interviews | Were repeat interviews carried out? If yes, how many? | No repeat interviews were carried out. |
| Audio/visual recording | Did the research use audio or visual recording to collect the data? | Audio recordings of each interview were collected. |
| Field notes | Were field notes made during and/or after the interview or focus group? | Field notes were made after each call to guide interpretation of interview coding. |
| Duration | What was the duration of the interviews or focus group? | Range: 21 to 77 minutes  Mean: 60 minutes |
| Data saturation | Was data saturation discussed? | Data saturation was discussed in the methods section, lines 131-132. |
| Transcripts returned | Were transcripts returned to participants for comment and/or correction? | Transcripts were not returned to participants. |
| **Domain 3: analysis and findings** | | |
| *Data analysis* | | |
| Number of data coders | How many data coders coded the data? | Three coders coded the data. |
| Description of the coding tree | Did authors provide a description of the coding tree? | The description of the coding tree is provided in Supplemental File 2. |
| Derivation of themes | Were themes identified in advance or derived from the data? | The codebook was based on the interview guide, but |
| Software | What software, if applicable, was used to manage the data? | NVivo 11 was used to manage the data |
| Participant checking | Did participants provide feedback on the findings? | Participants did not provide feedback on the findings. |
| *Reporting* | | |
| Quotations presented | Were participant quotations presented to illustrate the themes / findings? Was each quotation identified? e.g. participant number | Participant quotations were presented in the results section to illustrate themes. |
| Data and findings consistent | Was there consistency between the data presented and the findings? | Yes; the results in Table 1 are reflected in the narrative. |
| Clarity of major themes | Were major themes clearly presented in the findings? | Major themes are clearly presented in the narrative. |
| Clarity of minor themes | Is there a description of diverse cases or discussion of minor themes? | Diverse cases are not specifically discussed. |
